# On the Difference of the Respiratory Movements at Different Ages, Etc.

**Published:** 1843-11

**Authors:** 


*On the Difference of the Respiratory Movements at Different Ages, etc.*—We are not aware that the phenomena, to which Beau and Maissiat allude in the following remarks, have been sufficiently dwelt upon in any of the recent works on physiology; and yet they are of that practical importance as to merit the attentive consideration of all medical men. The gist of their observations is that the type of the respiratory movements, or, in other words, the manner in which these are performed, varies not a little in the two sexes, and also at the different periods of life. The following abstract of their elaborate memoir will enable our readers to judge of its contents.

They describe what they call three distinct types of the respiratory movements, viz: 1, the abdominal type; 2, the inferior costal type; and 3, the superior costal type. In the *first*, the cavity of the chest is increased during inspiration, chiefly by the descent of the diaphragm and the protrusion of the abdominal muscles, the ribs being nearly motionless;—in the *second* by the elevation and expansion of the lower six ribs, the movement of the upper ones becoming less and less considerable as we ascend, till it ceases altogether in the second and first;—and in the *third* chiefly by the elevation and expansion of the upper ribs, the movement becoming less and less marked as we descend, till it nearly ceases entirely in the lowermost. In the second variety, the lower half of the sternum is much more moved, *i. e.*, elevated and depressed, than its upper half; while the reverse of this is the case in the third variety.

In the majority of cases, each individual has, what may be called, his peculiar type of respiratory movements; and this seems to be remarkably constant at all times. One person breathes chiefly by the abdomen, as to speak; another by the lower ribs; and a third by the upper ones. A good deal however depends on age, and not a little on sex also, as we shall now endeavour to explain.

In the early period of life, and often indeed until three or four years of age, the respiration usually exhibits the abdominal type: the ribs are scarcely moved at all; and when the breathing is very much oppressed, as in many cases of pneumonia, the cartilages of the lower ribs are observed to be drawn somewhat inwards during each act of inspiration.

After this period of life, the three types begin to be marked in a more or less distinct manner, according to the individual's sex and constitution. The superior costal type is observed particularly in girls; while the other two types are found in nearly eqal frequency, in boys. As a person advances in life, we find that the first-mentioned type becomes more and more predominant in females; and one one of the latter types more and more so in males.

The superior costal type in the female sex is readily recognisable not only in our hospitals, but in any assemblies of women, and more especially in actresses on the stage. Who has not observed that the upper part of the thorax, including the sternum and clavicle, is often, during the representation of strong mental emotions, most strikingly elevated and depressed? Nothing at all similar is ever witnessed in male performers. It may be supposed that this peculiarity in women is in some degree induced by the wearing of stays and the consequent compression of the abdomen and the lower part of the chest. But, although this absurd and most pernicious custom* may unquestionably increase the degree or amount of the peculiarity in question, it certainly does not give rise to it; for we find it to exist in girls before they ever wore stays, and in countrywomen who never wear them at all.†

One important end gained by the respiration being superior-costal in the female sex is abundantly obvious—the breathing is thus much less disturbed than it otherwise would be during the period of pregnancy. This point, although seldom or never alluded to in modern lectures or writings, has not escaped the attention of some of the older physiologists. Boehaave, in his Prælectiones, thus makes mention of it: "Hinc in fæminis inspirantibus sternum rursum et oblique extrorsum vertitur, totusque thorax quasi assurgit; hinc etiam tumente abdomine, liberius respirant." Haller, the great commentator of Boerhaave, is still more exact and minute in his description of the peculiarities of the respiratory movements in the two sexes: "Considerate puerum anni unius et puellam ejusdem ætatis in eodem lecto unâ dormientis, videbis

---

* To do all manner of justice to the ladies, we must frankly admit that our authors are by no means so much opposed to the use of corsets as most medical men are. "We may here state *en passant*," they say, "that the use of the corset is not nearly so hurtful to females, as is generally imagined. Indeed it may almost be asserted that they are, so to speak, organised for the wearing of this piece of dress (!) On the other hand, it is an absolute physiological absurdity, (*contresens*), for men to wear stays; for their breathing is chiefly by the lower ribs and the abdomen."

† The eye is no guide in this matter, deceived as it must be, by the heaving of the mammæ, and the contiguous cellular and adipose tissues in the female, by which the movements of the chest are more visible than in the male sex; but it does not follow that they are really more active.—Ed. Bull. Med. Science.

in puella, quando inspirat, totam thoracis molem ascendere versus jugulum; in puero verò inspirante thoracem et claviculus vix moveri. In viro adulto pectus vix movetur equidquam, dum respirat; in fæminâ totum sursum trahitur, ut â diaphragmate recedat. Ergo vir abdomine maxime respirat, fæmina thorace. Nisi hanc fæmina diversitatem natura fecisset, gravidæ perpetuâ dyspnœâ laboravissant æque ac viri hydropici."

It would seem that most of the authors of the present day have quite overlooked these interesting peculiarities in the phenomena of the respiratory movements, so well described almost a century ago. It has been generally supposed that the movements are nearly alike in all persons; and hence one of the chief objects of inquiry among modern physiologists has been to determine the *where* and in what direction the principal enlargement of the chest takes place. We cannot therefore be surprised at the difference of opinion on this point. While Gerdy maintains that the greatest enlargement of the thoracic cavity occurs in a transverse direction on the level of the lower ribs, we find Hourmann and Dechambre infer from their observations—on the old female inmates of the Salpetriere hospital—that it is in the upper part of the chest, and that, "in this general movement of ascension, the sternum is, on an average, raised from eight to twelve lines." And what is the general conclusion to which they come? That "in proportion as a person advances in years, the motory powers of respiration—and more especially those which tend to increase the transverse dimensions of the chest—become considerably impaired in their energy." It is scarcely necessary to say that the mode of respiration, described by these gentlemen, is not the result of age, but is natural to the female sex. It is however much more conspicuous in young than in old women.

Beau and Maissiat proceed to notice the peculiarities of the respiratory movements in different animals; for the type, so to speak, is far from being alike in all. In the rabbit, the horse, the cat, &c., it is chiefly abdominal; while in the dog, it is inferior costal in a very marked degree. In no animal is it superior costal, at least in the natural and healthy condition; and perhaps for this reason that the alternate elevation and depression of the upper ribs and of the sternum would have interfered not a little with the movements of the fore legs in running. This mode of respiration may therefore be regarded as peculiar to the human race, and more especially to the female sex.—*Bulletin Med. Sci.*, from *Encyclographie des Sciences Medicales.*

